# A Network Flow-based Analysis of Cognitive Reserve in Normal Ageing and Alzheimer’s Disease

**DOI:** 10.1038/srep10057

**Published:** 2015-05-20

**Authors:** Sang Wook Yoo, Cheol E. Han, Joseph S. Shin, Sang Won Seo, Duk L. Na, Marcus Kaiser, Yong Jeong, Joon-Kyung Seong

**Affiliations:** 1 Department of Biomedical Engineering, Korea University, Seoul, Republic of Korea; 2 Department of Computer Science, KAIST, Daejeon, Republic of Korea; 3 Handong Global University, Pohang, Republic of Korea; 4 Department of Neurology, Sungkyunkwan University School of Medicine, Samsung Medical Center, Seoul, Korea; 5 Department of Brain & Cognitive Sciences, Seoul National University, Seoul 151–747, South Korea; 6 Interdisciplinary Computing and Complex BioSystems Research Group, School of Computing Science, Newcastle University, Newcastle upon Tyne, NE1 7RU, UK; 7 Department of Bio and Brain Engineering, KAIST, Daejeon, Republic of Korea

## Abstract

Cognitive reserve is the ability to sustain cognitive function even with a certain amount of brain damages. Here we investigate the neural compensation mechanism of cognitive reserve from the perspective of structural brain connectivity. Our goal was to show that normal people with high education levels (i.e., cognitive reserve) maintain abundant pathways connecting any two brain regions, providing better compensation or resilience after brain damage. Accordingly, patients with high education levels show more deterioration in structural brain connectivity than those with low education levels before symptoms of Alzheimer’s disease (AD) become apparent. To test this hypothesis, we use network flow measuring the number of alternative paths between two brain regions in the brain network. The experimental results show that for normal aging, education strengthens network reliability, as measured through flow values, in a subnetwork centered at the supramarginal gyrus. For AD, a subnetwork centered at the left middle frontal gyrus shows a negative correlation between flow and education, which implies more collapse in structural brain connectivity for highly educated patients. We conclude that cognitive reserve may come from the ability of network reorganization to secure the information flow within the brain network, therefore making it more resistant to disease progress.

Cognitive reserve refers to the brain’s ability to cope with increasing brain damage or age-related degeneration while still functioning appropriately. This concept originates from the repeated observation of the inconsistency between the severity of brain pathology and the clinical deterioration^1^. For example, Katzman *et al.*^2^ observed advanced pathology of Alzheimer’s disease (AD) from brains of ten cognitively normal people at their death. In addition, the Nun Study showed that more highly educated nuns had fewer symptoms of cognitive decline^3^. These observations imply that normal people with the same cognitive function may have different levels of brain pathology according to the amount of their cognitive reserve.

It is known that the difference of cognitive reserve between individuals is related to the lifelong experiences including educational or occupational attainment, and leisure activities in later life^4^. Education levels have been widely used as a measure for educational attainment as it is direct and easy to obtain. Epidemiological studies found that individuals with lower education levels had higher risk of developing AD^5^, while individuals with higher education levels showed less chance of developing AD, however, more rapid decline of cognitive function when they got AD^6^,^7^. This difference of educational effects between normal people and AD patients has been attributed to the individual difference in the ability to maximize the cognitive performance through differential recruitment of brain resources or use of alternate networks during the course of brain degeneration^1^.

There are two hypothesized components underlying cognitive reserve: neural reserve and neural compensation; the former concerns individual differences of neural efficiency and capacity in normal aging, and the latter deals with that in the ability to compensate for brain damage^4^. Functional imaging studies have tried to explain these mechanisms by observing the changes of task-related activation patterns according to the severity of AD pathology^8^,^9^,^10^,^11^. Studies using resting regional cerebral blood flow (rCBF) showed that patients matched for clinical severity had a negative correlation between resting rCBF and education levels^12^. A study using positron emission tomography (PET) demonstrated that the early-onset AD patients with presumably higher cognitive reserve had more hypometabolic areas than the late-onset AD patients with similar clinical severity^13^. These studies speculated on the existence of cognitive reserve by showing lower resting rCBF or higher task-induced activity in individuals with high reserve, but it is still challenging to provide direct and quantitative neural measures of cognitive reserve^4^.

There have been several attempts to quantify the cognitive reserve based on brain images using hippocampal volume^14^, total brain volume, regional gray matter volume^15^, or regional cerebral blood flow^16^ as surrogate marker. The most plausible measure for cognitive reserve is the Blood-oxygen-level dependent (BOLD) signal change during certain cognitive tasks^17^,^18^,^19^,^20^. In these tasks, an increase in activation with an increase in cognitive demand is assumed to reflect cognitive reserve. Stern *et al.* introduced the concept of brain networks that are associated with cognitive reserve by comparing healthy young and old adults^21^: This study used years of education and scores of cognitive function tests as indices for cognitive reserve, and found different topographic patterns during a serial recognition task. However, so far none of these studies have adopted topological brain network features as measures of cognitive reserve.

The primary goal of this study was to investigate cognitive reserve from a network perspective, by extracting white matter (WM) networks from diffusion-weighted magnetic resonance images (MRIs) and observing how the WM network connectivity changes with education levels. For WM brain network analysis, we adopt a novel measure for network connectivity based on the concept of maximum flow. In graph theory, the maximum flow from a source node to a sink node in a graph represents the maximum transportation capacity from the source to the sink through the paths connecting these nodes in the graph. In case of a binary-weighted graph, a large maximum flow value from a source node to a sink indicates the existence of many alternative paths that connect the two nodes. This means that their connectivity is robust and reliable so that information can still flow between them even when certain paths are disconnected. In this sense, the individual difference in the maximum flow values can be assumed to have a tight relationship with the cognitive reserve, that is, the ability of compensation or resilience to the brain damage. Recently, a similar network measure, called communicability, has been introduced to detect network changes after stroke^22^,^23^. The communicability between two nodes counts the number of paths between them, which may not reflect the actual information flow in the WM brain networks as it allows an edge to be involved in several paths repeatedly.

We hypothesized that people with higher cognitive reserve would have higher maximum flow in WM brain networks. In order to demonstrate this hypothesis, we analyze correlation between the education levels and the maximum flow values in normal control (NC) and AD patient groups separately. We recruited 80 AD patients and 80 NC subjects of which age and gender are matched. For both groups, education levels were measured as total duration of formal education, which ranged from 0 to 22 years. Figure 1a shows a schematic overview of our hypothesis. In the case of positive correlation, we speculate that education indeed strengthens the WM connectivity of certain subnetworks, which has more alternative routes between two nodes. On the other hand, negative correlation implicates that the WM connectivity of certain subnetworks has been disrupted in subjects with higher education levels. This can facilitate the quantitative analysis on cognitive reserve from a brain network perspective.

## Results

### Maximum flow: A reliability measure for brain connectivity

For a binary-weighted graph, the maximum flow value is equal to the number of edge-disjoint paths between source and destination nodes in the graph^24^. Supplementary Fig. 1c shows an illustrative example. Two binary graphs have the same number of edges but different maximum flow values from node *i* to node *j*. There exist two edge-disjoint paths from node *i* to node *j* for the left graph, while four paths exist for the right one. Hence from the perspective of reliability or robustness in information flow, the right graph is more resistant to possible destruction of edges.

This idea was tested with the WM networks constructed using MRI of subjects in the NC (*n* = 80) and AD (*n* = 80) groups. For each subject, the WM network was constructed with the whole brain regions as nodes, and a maximum flow value was computed for every pair of nodes in the network (See Supplementary Methods section for details of the maximum flow computation method). Figure 2a shows ordered lists of node pairs in terms of the maximum flow values between two nodes for the binary WM brain networks of the NC and AD groups, respectively. The figures in each row show the top 50 node pairs from three different views, and the table following the figures shows 5 node pairs with the largest maximum flow values. The 5 top node pairs all involve the precuneus. The connected nodes are left middle temporal gyrus and putamen in NC, and putamen and calcarine gyrus in AD, respectively. The resulting rankings in the two groups are fairly consistent, indicating substantial overlap of brain regions with larger maximum flow values regardless of the groups. However, the degree of network reliability represented by the maximum flow value is significantly smaller for the AD group: The average flow values of each subject were compared between two groups after statistically controlling for the effect of age, gender, and education levels (ANCOVA, p-value < 0.0001). Also, Fig. 2b shows another result of a group comparison of the maximum flow values for each pair of nodes in the networks. We performed an analysis of covariance (ANCOVA), after statistically controlling for the effect of age, gender, and education levels. For multiple comparisons correction, we used the Bonferroni method^25^. Figure 2b displays a set of node pairs with the corrected p-values less than 0.05. There exist 100 such node pairs after correction, and the table lists the top 10 node pairs with the most significant group difference. The node pairs with significant group difference include those that have the greatest maximum flow values such as a connection between precunus and putamen.

### Relationship between maximum flow and education levels

**Figure 1:**
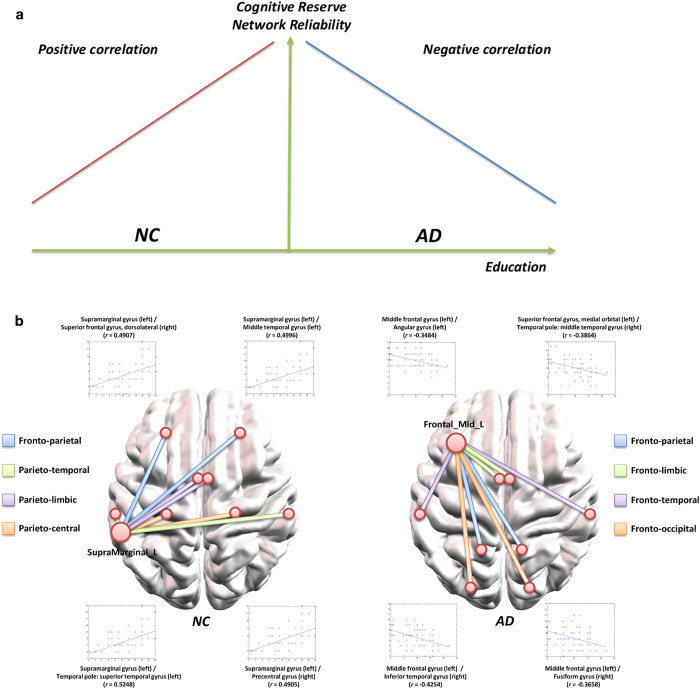
(**a**) A schematic overview of the cognitive reserve hypothesis: In the case of positive correlation (normal aging), we speculate that education indeed strengthens the WM connectivity of certain subnetworks, which has more alternative routes between two nodes. On the other hand, negative correlation implicates that the WM connectivity of certain subnetworks has been disrupted more in subjects with higher education level. (**b**) For normal aging, network reliability has positive correlation with education levels, specifically in the subnetwork with a core node at the left supramarginal gyrus. For AD, network reliability has negative correlation with education levels, specifically in the subnetwork with a core node at the left middle frontal gyrus.

**Figure 2:**
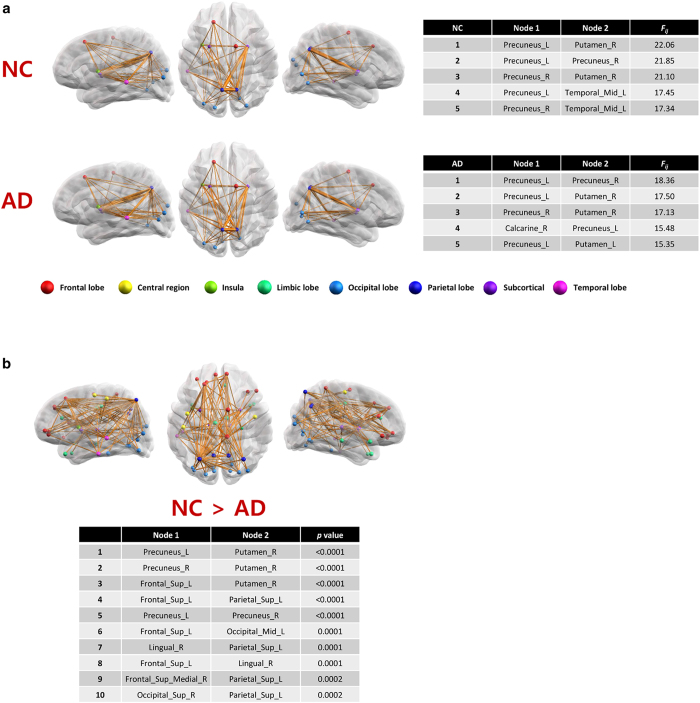
An example of maximum flow computation and group comparison. (**a**). The maximum flow was computed for every pair of nodes and thus every edge in the WM network has a maximum flow value. The edges in the WM network are sorted in terms of the maximum flow values for both NC and AD groups separately. Each row in the figure shows the top 50 edges and the table lists 5 edges with the largest maximum flow values. (**b**). This figure shows the result of a group comparison of the maximum flow values for each pair of nodes in the networks. After edge-by-edge comparison between two groups, the figure shows a set of edges of which maximum flow values are significantly different (corrected p-value < 0.05). The table lists top 10 edges with the most significant group difference.

Represented by the maximum flow values, the number of edge-disjoint paths between a pair of nodes in each subject could be correlated with education levels in a positive or negative way. For each subject, we measured the education level as the duration of formal education, which could be ranged from 0 to 22 years. Figure 1a shows a schematic graph for the relationship between the maximum flow values and education levels. Suppose that the maximum flow values of the subjects between nodes *i* and *j* have a high positive correlation coefficient with their education levels for the NC group. Then, the connection (*i,j*) tends to be more reliable with higher education, which in turn implies that education strengthens the reliability of brain connectivity between the two nodes. Such a connection therefore plays a key role in maintaining normal cognitive functions even with more brain damage. Similarly, we can also postulate that a negative correlation for a connection in the AD group indicates more damage in WM connectivity for highly educated subjects. As shown in Fig. 1a, negative correlation in AD implies that the reliability of the network connection *(i,j)* is reduced more for highly educated AD patients with similar levels of cognitive impairment.

To demonstrate the relationship between cognitive reserve and brain network reliability, we analyzed the correlation between education levels and the maximum flow values of subjects in the NC as well as in the AD group. For ease of reference, we use the flow value (of a subject) for a pair of nodes to refer to the maximum flow value (of the subject) between two nodes. Similarly, a correlation coefficient (of all subjects) for a pair of nodes is used to refer to a positive (or negative) correlation coefficient (of all subjects) between education levels and the maximum flow values between two nodes. As explained in the Network analysis section, we seek a subnetwork of the WM networks such that the maximum flow values have a statistically significant correlation with education levels in a positive or negative way.

### Education strengthens brain network reliability in normal aging

**Figure 3:**
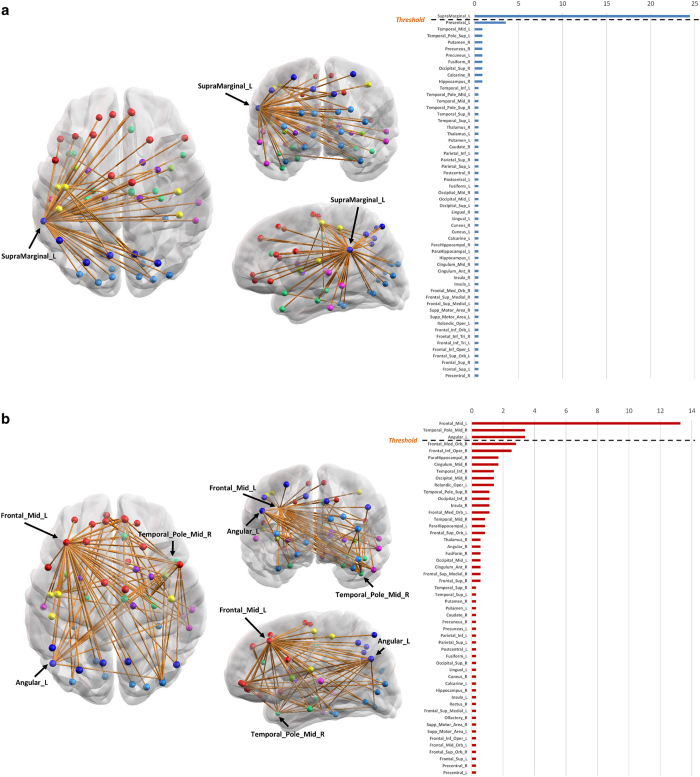
3D representations of subnetworks (**a**). 3D representations of a subnetwork of the NC group which has the significant positive correlation between education level and maximum flow. (Fiber number threshold = 3, correlation threshold = 0.32, p = 0.026 ± 0.004, cluster size = 63) (**b**). 3D representations of a subnetwork of the AD group which has the significant negative correlation between education level and maximum flow. (Fiber number threshold = 3, correlation threshold = −0.3, p = 0.041 ± 0.006, cluster size = 92).

For subjects of the NC group, we identified a subnetwork that represents a set of connections (or regions) that were affected by the education levels in a positive way. In other words, the identified subnetwork consists of edges such that the maximum flow values for all subjects in the group from a vertex of each edge to the other have a positive correlation with education levels: For the NC group, there exists no subnetwork that has a significant negative correlation between the maximum flow values and education levels. The resulting subnetwork is listed in Supplementary Table 1. Statistical significance of the subnetworks was measured based on the suprathreshold cluster size test (See Network analysis section for details). The subnetwork consists of 56 nodes and 63 edges. The correlation threshold was 0.32, and the *p*-value interval was 0.026  ±  0.004. The confidence interval for the *p*-value was estimated parametrically as given in the work by Zalesky^26^. A three-dimensional visualization of the subnetwork is shown in Fig. 3a. The identified subnetwork predominantly comprises connections in fronto-parietal, parieto-temporal as well as parieto-limbic and parieto-central connections, which were known to be involved in various cognitive functions such as language, episodic memory retrieval and so on^27^. With higher education levels, information flow becomes more reliable through the alternative routes. The subnetwork was centered at the left supramarginal gyrus, which was also identified as a network hub with the highest centrality value for node betweenness centrality and closeness centrality in the resulting subnetwork (See Supplementary Methods section for details).

### Brain network reliability negatively correlates with education in AD

For patients with AD, we identified a subnetwork that represents a set of connections (or regions) that were affected by the education levels in a negative way. The subnetwork for the AD group consisted of 52 nodes and 92 edges. The correlation threshold was −0.3, and the *p*-value interval for the subnetwork was 0.041 ± 0.006. Supplementary Table 1 lists the nodes of the subnetwork as well as their degrees in the subnetwork. Figure 3b shows a three-dimensional visualization of the subnetwork. As shown in Supplementary Table 1 and Fig. 3b, the identified subnetwork predominantly comprises fronto-frontal and fronto-parietal as well as fronto-limbic, fronto-temporal, and fronto-occipital connections. Hubs of the subnetwork were the left middle frontal gyrus, the right middle temporal pole gyrus, and the left angular gyrus (see Supplementary Table 1). This finding supports the previous findings that showed recruitment of frontal areas in early AD^28^,^29^. While the subnetwork is bilateral, the left hemisphere is clearly more correlated with education levels. Indeed, the node with the greatest number of connections was the left middle frontal gyrus, which is related to the executive function, decision making and logical thinking^30^. A negative correlation was also observed in another association cortex centered on the left prefrontal areas. For the AD group, there exist no subnetwork having a significant positive correlation between the maximum flow values and education levels.

### Group comparison of educational effects

In the above experiments, we found two subnetworks in binary WM networks for the NC and AD groups, respectively, based on the network flow. The subnetwork identified for the NC group showed positive correlation between the maximum flow values and the education levels: the average correlation coefficient for all edges in the subnetwork was 0.44. For the same subnetwork, we analyzed such correlation using the subjects in the AD group. The average correlation coefficient was −0.079, which implies that the maximum flow values in the subnetwork were not correlated significantly with the education levels in AD. We did the same analysis for the subnetwork identified in the AD group using the subjects in the NC group. The result showed the average correlation coefficient equals to −0.034, therefore indicating no significant correlation.

In addition to the within-group educational effect, we also investigated group difference of the educational effect. We first constructed a correlation matrix for each group, of which an entry is the correlation coefficient between the maximum flow value and the education level for an edge. We then compared the correlation coefficients between two groups for every pair of nodes. We found that the correlation coefficients in the AD group were significantly smaller than those of the NC group for several edges in the WM network (Supplementary Table 2). Great differences were observed in the connections from the left supramarginal gyrus, which was identified as a hub node. All the connections identified in this experiment were contained in the statistically significant edges in the subnetworks. Moreover, the nodes with the greatest degree were also coincided with the hub nodes of the subnetworks. This finding further supports the idea that the subnetwork centered at the supramarginal gyrus play a key role for the cognitive reserve.

### Network summary measures

We next examined the relationships between the network summary measures (total number of edges, total number of neural fibers, sum of fractional anisotropy (FA) values, and sum of maximum flow values) and education levels. For each subject, we measured the total number of edges, the total number of streamlines, the sum of FA values, and the sum of the maximum flow values in the WM network. We then performed generalized linear regression^31^ to investigate their relationships with education levels. Age and gender were taken as covariates in this model. The results are listed in Table 1. In both groups, all of the measures showed no significant correlation with the education levels.

### Reproducibility of findings

To test the reproducibility of our results, we repeated the subnetwork identification for randomly generated sub-groups of AD and NC group data. For the NC group, we generated 20 sub-groups with age, gender, and education level matched for each of the NC and AD groups by randomly removing 10% of the subjects in the group. We further matched the Clinical Dementia Rating-Sum of Boxes (CDR-SOB) score to generate 20 random sub-groups of the AD group. For each sub-group, there were no significant correlations of age and gender (also CDR-SOB score for AD group) with the levels of education (*p* > 0.05 for all cases). We performed the suprathreshold cluster size test for each sub-group independently. The correlation threshold was set to the value that showed consistent subnetworks across different edge construction thresholds (*r* = −0.3 fo*r* the AD group, *r* = 0.32 fo*r* the NC group). For the AD group, the reproducibility experiments exhibited that the results were statistically significant for 18 among 20 different random sub-groups, and these 18 subnetworks contained 88.6% of the network connections extracted from the original experiment on average. For the NC group, 18 sub-groups showed significant results, which contained 85.3% of the network connections identified by the original experiment on average.

We also tested the influence of different edge construction thresholds on subnetwork identification. In our experiments, the binary WM networks were constructed with three fibers as the threshold. However, it is well-known that the choice of a threshold value affects the topological organization of the resulting binary network^32^. In this reproducibility analysis, we evaluated the effect of different thresholds on network analysis by varying the threshold from 1 to 5. We found that these threshold values did not significantly influence on our results (Table 2).

## Discussion

In this study, we investigated cognitive reserve in normal aging and AD within the framework of network flow in graph theory. We postulated that the cognitive reserve is related to the maximum flow values between nodes in certain subnetworks. We identified a subnetwork for the NC group that has positive correlation between the maximum flow values and education levels and another subnetwork for AD patient group that has negative correlation between the maximum flow values and education levels. The subnetwork is centered at the left supramarginal gyrus for the NC group and at the left middle frontal gyrus for the AD group. Analyzed separately for both groups, the results strongly suggest specific neural mechanisms of cognitive reserve (Fig. 1a).

To the best of our knowledge, the cognitive reserve hypothesis has not been dealt with from the network perspective. Previous studies focused on the quantitative concept called brain reserve, which states that the number of neurons or synapses are different across individuals with different education levels^4^,^33^,^34^. For example, neuroimaging studies showed that highly educated subjects with normal cognition had more brain volume than poorly educated subjects, while highly educated subjects with AD showed more hypometabolism^35^ and cortical atrophy^34^ than poorly educated subjects with AD. In contrast to the quantitative nature of brain reserve, cognitive reserve is an active form of reserve that shows how well the brain is functioning^1^. Epidemiological or functional MRI studies provided various evidences that support the cognitive reserve hypothesis. However, epidemiological approaches lack in identifying localized regions that are related to the cognitive reserve. Although several neuroimaging studies investigated surrogate markers to quantify cognitive reserve, including hippocampal volume^14^, regional gray matter volume^15^, regional cerebral blood flow^16^, these works are limited in providing quantitative measures of neural compensation mechanism of cognitive reserve. Functional MRI studies were also restricted to certain specific tasks. In our study, we investigated WM brain networks to identify specific brain connectivities that mediate the cognitive reserve.

The maximum flow value for a pair of nodes in our binary WM network implies the reliability of their connection in the brain network of a subject. Both NC and AD group showed the highest maximum flow value centered at precuneus. This result is in line with the idea that the precuneus is one of the hub brain regions and also a core structure of the default mode network^36^,^37^. From the clinical perspective, this value is regarded to represent how robust the connectivity for the nodes is when a disease progresses. The reliability of the network was often investigated through random or targeted attack experiments^38^. Attacks on the edges may be related to the deterioration in the white matter by focal ischemia or degenerative processes. Disconnected edges hinder information flow by increasing the shortest path length between nodes. Similarly, attacks on nodes, in particular, removal of the hub nodes of high degree leads to a critical problem such as fragmented networks. However, even in the case of disconnected edges or damaged nodes, human brains which have high connectivity could still perform their functions if there exist alternative routes bypassing the problematic nodes or edges. Accordingly, we speculate that brain networks with more alternative routes between nodes provide more ability of compensation or resilience to brain damages. The maximum flow value for a pair of nodes in a binary WM network captures how many edge-disjoint routes exist between the two nodes, which enables us to estimate the tolerance of a brain to perturbation from diseases.

The connectivity of the subnetwork for the NC group tended to be reliable (strong) for educated subjects. The subnetwork consisted of parieto-frontal and parieto-temporal, as well as parieto-limbic and parieto-central connections. The hub node was located at the parietal cortex, specifically left supramarginal gyrus (Supplementary Table 1). However, the connectivity of this subnetwork was not positively correlated with education levels for the AD group, which could mean that it was deteriorated before clinical presentation of AD. On the other hand, the connectivity of the other subnetwork was negatively correlated with education levels for the AD group, which could imply that it was degraded more rapidly for educated AD subjects. The subnetwork predominantly comprises connections in fronto-frontal and fronto-parietal, as well as fronto-limbic, fronto-temporal, and fronto-occipital. The hub brain region is located at the frontal area, specifically the left middle frontal gyrus (Supplementary Table 1). These results further support the existence of the hypothesized neural mechanisms for cognitive reserve.

Given the above observations, we postulate that cognitive reserve may be based on the ability of network reorganization to secure the information flow within the brain network. The existence of multiple alternative routes could be interpreted to have neural compensation or back-up plans for the brain. As the brain network degenerates, the default pathway is breaking down. To compensate and to maintain the information flow, an alternative second best pathway will replace the original pathway. Thus, the network is more reliable (robust) to sudden damage or disease progression if there are more alternative pathways. Education and other social activities help the development of alternative routes and therefore facilitate functional compensation if needed later on.

### Methodological limitations

Our method has the following limitations. First, it is not possible to show rapid disruption of WM network connectivity in individuals with higher cognitive reserve because we did not perform a longitudinal study. However, we believe that a cross-sectional study with a large number of subjects of different education levels and different cognitive deficits would already indicate characteristic changes. Second, we only used education levels as a factor that affects cognitive reserve. Although the cognitive reserve includes effects of other factors such as occupation and social activities, we only employed the education duration because the others are hard to quantify. Third, the deterministic tractography which we used to construct network edges may lose crossing fibers. Alternatively, probabilistic tractography^39^ may be used. Fourth, as noted in the paper (Han *et al.*, 2013) introducing cluster-based statistics for a correlation analysis of network edges, selection of initial thresholds can affect the result. Following this paper, we systematically searched initial thresholds and confirmed the stability of the results. Finally, we found that our method shows a high reproducibility for different data as described in the Reproducibility analysis section. Although clusters of the random sub-groups were similar to that of the original cluster, there were small variations in the nodes of clusters. The variations were probably due to the noise effect of correlation analysis. This noise effect will be smaller as more data are used in correlation analysis.

### Future work

In the current study, we only used binary networks because of their simpler interpretation. The maximum flow between two nodes in a binary network represents the number of distinct paths between them. However, fiber number (FN) or FA networks can also be analyzed with appropriate interpretations. In the future, we would further study those different networks. Moreover, we may perform this analysis for different parcellation schemes as node definition can affect network properties^36^,^40^,^41^,^42^,^43^. We could also perform the analysis for patients with other cognitive impairment such as mild cognitive impairment, frontotemporal dementia and dementia with Lewy bodies. Although we only used maximum flow values for WM brain network analysis, the edges in a minimum cut set also provide useful information. A minimum cut set represents a bottleneck in the information flow of a brain system, and thus the cognitive process of the system could be disrupted with the impairment of edges in this set due to a neurological disease. Finally, a longitudinal study would be vital to demonstrate progressive alterations of brain networks in AD.

## Conclusion

In conclusion, the results support the existence of hypothesized neural mechanisms for cognitive reserve based on network flow analysis of binary WM brain networks. The maximum flow is centered at the hub region, precuneus in both AD and NC groups. We identified two subnetworks of the WM brain networks that might represent cognitive reserve. The subnetworks for the AD group were mainly composed of connections in fronto-frontal, fronto-parietal, fronto-limbic, fronto-temporal, and fronto-occipital areas, of which the robustness of connectivity had a negative correlation with education levels. This finding supports the cognitive hypothesis that cognitive functions are more severely impaired in AD patients with higher education levels. The subnetwork for the NC group was mainly composed of connections in parieto-temporal, parieto-frontal, parieto-limbic, and parieto-central areas of which the robustness of connectivity had a positive correlation with education levels. This also provides evidence to support the cognitive reserve hypothesis. The maximum flow can be used in the assessments of robustness or resilience of each individual and further used as a biomarker for prediction of developing dementia, prognosis and also for monitoring the response to drugs or other interventions.

## Methods

### Subjects

We recruited 80 patients with AD and age, gender and education level-matched 80 NC subjects at the Samsung Medical Center in Seoul Korea. The demographic characteristics of the subjects are presented in Table 3. We obtained written consent from each patient and his/her caregiver, and the Institutional Review Board of the Samsung Medical Center approved the study protocol. The study was carried out in accordance with approved guidelines. NC subjects had no history of neurological or psychiatric illnesses other than headache or dizziness and had no memory complaints and performed normally on neuropsychology tests. AD patients fulfilled the criteria for probable Alzheimer’s disease proposed by the National Institute of Neurological and Communicative Disorders and Stroke and the Alzheimer’s Disease and Related Disorders Association (NINCDS-ADRDA)^34^,^44^,^45^,^46^. All patients completed a clinical interview and neurological examination as described previously^47^ and underwent neuropsychological testing using the Seoul Neuropsychological Screening Battery (SNSB)^48^. This screening contains quantitative tests, including Korean version of MMSE (K-MMSE) and CDR-SOB.

To remove the effect of age and gender (also CDR-SOB score for the AD group) on the correlation coefficients, we performed partial correlation analysis^49^. There were no significant correlations of the education levels with age, gender, and CDR-SOB score in both groups. Specifically, the AD group has the correlation coefficient of r = 0.021 (p = 0.85), r = −0.032 (p = 0.78), and r = 0.138 (p = 0.22) for the age, gender, and CDR-SOB score, respectively. Similarly, the group of NC has r = −0.090 (p = 0.43) and r = −0.188 (p = 0.09) for the age and gender, respectively. Also, for the NC group, all subjects have zero CDR-SOB score except for 15 subjects (the average CDR-SOB score is 0.16). There were no significant differences in age (p = 0.12) and education levels (p = 0.16) between the AD and NC groups. However, there were significant differences in MMSE scores (p = 2.27 × 10^−33^) and CDR-SOB scores (p = 1.01 × 10^−25^) between the two groups. For all subjects in both groups, to evaluate the level of education achieved by participants precisely, we inquired in detail about their formal education, including whether or not they had completed each step of education (elementary school, middle school, high school, college, and graduate school) and total duration of education. Table 3 summarizes the results of statistical analysis on the demographics.

### Data Acquisition

T1 and diffusion-weighted MRIs were acquired from all 160 subjects using 3.0 T MRI scanner (Philips 3.0T Achieva). T1-weighted MRIs were obtained using the following parameters: axial slice thickness = 5.0 mm; inter-slice thickness = 2 mm; TR = 669 ms; TE = 16 ms; flip angle = 18 °; matrix size = 560 × 560. In the whole-brain diffusion-weighted MRIs, sets of axial diffusion-weighted single-shot echo-planar images were obtained using the following parameters: TR = 7383 ms; TE = 60 ms; flip angle = 90 °; field of view = 22 × 22 cm^2^; matrix size = 128 × 128; voxel size = 1.72 × 1.72 × 2 mm^3^; slices = 70; slice thickness = 2 mm; b = 600 s/mm^2^. With the baseline image without weighting (b = 0), diffusion-weighted images were acquired from 45 different directions. All axial sections were acquired parallel to the anterior commissure-posterior commissure line.

### Network construction

We model a human brain as a network called a WM brain network, which is represented as an undirected graph, *G = (V, E, W)*, where *V* is a set of nodes, *E* is a set of edges connecting the nodes, and *W* is a set of weights associated with the edges. In general, three types of edge weights have been used in the WM network: binary values (zero or one), mean FA values, and numbers of fiber tracts connecting nodes. The binary weight of an edge represents existence (one) or absence (zero) of the connection between two nodes. FA is known to be closely related to fiber integrity^50^,^51^. Since there is currently no consensus regarding the selection of weight values for quantifying structural connectivity with tractography measurements, various alternatives were exploited in constructing weighted structural connectivity networks, including FN, mean FA values, and binary values^52^,^53^,^54^,^55^. We adopt binary weights to measure the robustness (or reliability) of (alternative) connectivity between nodes in relation to education levels.

Supplementary Fig. 1a shows an overview of the WM network construction procedure. This procedure starts with identifying the cortical regions or sub-cortical structures with the automated anatomical labeling (AAL) template^56^. The AAL template contains 90 anatomical regions (78 cortical regions and 12 sub-cortical structures) as shown in Supplementary Table 3. We first linearly register the T1-weighted MRI of a subject to a b0 (reference) image in the diffusion MRI space, and then nonlinearly transformed the T1-weighted MRI to the ICBM152 T1 template in the MNI space where the AAL template regions are defined. To obtain AAL regions of a subject, we map the AAL atlas from the MNI space back onto the original T1 space using the inverse of the non-linear transformation followed by the linear transformation from the T1 space to the original diffusion MRI space. While mapping back the atlas, we use the nearest neighbor interpolation method^57^ to preserve the discrete labels of the AAL atlas. By considering each AAL region as a node, a total of 90 nodes are obtained automatically. We use the Linear Image Registration Tool (FLIRT)^58^ and the Non-linear Image Registration Tool (FNIRT)^59^,^60^ of the FMRIB Software Library (FSL) for the linear and non-linear registrations, respectively.

The edges between nodes are constructed with bundles of fiber tracts connecting the nodes. In order to extract the fiber tracts, we first corrected the eddy current distortions and the head motions in the diffusion-weighted MRIs of a subject by applying an affine transform of each diffusion-weighted MRI to the b0 image using FMRIB’s Diffusion Toolbox (FDT). Then, the diffusion tensor was estimated for every voxel from the corrected diffusion-weighted MRIs, and the FA of each voxel was also computed with the eigenvalues of the tensor. Finally, we employed a deterministic tractography algorithm^61^ to extract the fiber tracts. The tracking is initiated by seeding each voxel with an FA value greater than 0.2, and the tracking is stopped when the angle between the two last moves is greater than 45 degrees or when the tract reaches a voxel with an FA value less than 0.2^62^. The DTI-Studio^63^ was exploited for the diffusion tensor estimation, FA value computation, and tractography.

Shu *et al.* considered two nodes (regions) of the WM network to be linked through an edge if there is a fiber bundle containing three or more fiber tracts between these regions^57^. They showed that this threshold reduces the risk of false-positive connections due to noise or the limitations in the tractography. In order to construct the edges, we also choose the same threshold value for connecting two regions with an edge, which has a binary value of one as its weight. This WM network is represented as a 90 × 90 matrix for each subject.

### Network analysis

We first describe how to identify the subnetwork for the NC group. The proposed method consists of two steps: correlation coefficient computation and subnetwork construction. In the former step, for every pair of nodes, we compute the correlation coefficient between education levels and maximum flow values. Age and gender were used as covariates to remove their effect on correlation coefficient computation. As the result, we obtain a 90 × 90 matrix containing the correlation coefficients for all pairs of nodes. In the latter step, we perform the supra-threshold cluster size test^64^ on this matrix in order to identify the subnetwork and also compute its *p*-value. We first find the edges (and the corresponding pairs of vertices) with their correlation coefficients larger than a given threshold to form a (possibly disconnected) subgraph of the WM network and then find their connected components to obtain a collection of clusters. Each connected component corresponds to a cluster. Let the size of a cluster be the number of edges in the cluster. The maximum size over all clusters is used as the representative statistic, which is chosen as the subnetwork of the NC group. The *p*-value of this subnetwork is estimated with its size on the null distribution obtained by random permutations of the education levels of subjects. Specifically, we generate *M* permutation vectors by randomly permuting the education levels of subjects in a given group, and find the largest cluster for each permutation vector as described above. The cluster sizes for all permutation vectors form a null distribution of the maximum cluster sizes. When performing a set of statistical inferences simultaneously for all edges, the *p*-values of each edge should be corrected for multiple statistical inferences. The *p*-value of the subnetwork computed above reflects the multiple inference correction, and provides the statistical significance level for each edge in the subnetwork^65^,^66^. In the similar manner, we identify the subnetwork for the AD group and its *p*-value. The difference is that the CDR-SOB score is also used as a covariate in the correlation coefficient computation step to match the level of clinical severity across AD subjects. Also, we collect the edges with their correlation coefficients smaller than a given threshold.

The suprathreshold cluster size test has a number of issues including the followings: how to define spatial neighbors and how to determine the correlation threshold. The decision for each of these issues can greatly affect the outcome of the test^66^. The first issue can be naturally resolved since the adjacency of two node pairs can be defined based on the graph structure: Two node pairs are adjacent if they share a common node. For the second issue, it is difficult to provide definitive rules guiding how to choose the set of suprathreshold links^26^. If the threshold is chosen too low, large clusters result in permuted data as a matter of chance and thereby reduce the statistical power. In contrast, if the threshold is set too high, node pairs with high education effect may be excluded from the set of suprathreshold links. We therefore perform the suprathreshold cluster size test for a range of the thresholds. We then choose a correlation threshold which gives consistent subnetworks across the different thresholds.

## Author Contributions

Conceived and designed the experiments: S.W.Y., C.E.H., J.S.S., M.K., J.K.S. Performed the experiments: S.W.Y., C.E.H. Analyzed the data: S.W.Y., C.E.H. Contributed reagents/materials/analysis tools: S.W.Y., C.E.H. S.W.S., D.L.N. Wrote the paper: S.W.Y., C.E.H. J.S.S., Y.J., J.K.S.

## Additional Information

**How to cite this article**: Wook Yoo, S. *et al*. A Network Flow-based Analysis of Cognitive Reserve in Normal Ageing and Alzheimer's Disease. *Sci. Rep.* **5**, 10057; doi: 10.1038/srep10057 (2015).

## Supplementary Material

Supplementary Information

## Figures and Tables

**Table 1:** Coefficients between education level and network summary measures.

**Table 2:** Correlation threshold, p-value, and size of subnetworks for WM networks constructed with different fiber number thresholds in AD and NC groups.

**Group**		**Threshold = 1**	**Threshold = 2**	**Threshold = 3**	**Threshold = 4**	**Threshold = 5**
**NC**	***r*** **threshold**	0.32	0.32	0.32	0.32	0.32
	***p*****-value**	0.041 ± 0.006	0.025 ± 0.004	0.026 ± 0.004	0.038 ± 0.005	0.044 ± 0.006
	**Cluster size**	54	70	63	50	50
**AD**	***r*** **threshold**	–0.30	–0.28	–0.30	–0.30	–0.30
	***p*****-value**	0.019 ± 0.004	0.032 ± 0.005	0.041 ± 0.006	0.027 ± 0.005	0.044 ± 0.006
	**Cluster size**	121	151	92	101	80

**Table 3:** Demographic characteristics of AD and NC groups.

	**NC (80)**	**AD (80)**	***p*****-value**
Age	70.18 (5.99)	72.09 (9.09)	0.118
Sex (M/F)	38/42	37/43	0.875
Education level	11.33 (4.77)	10.18 (5.41)	0.158
K-MMSE	28.60 (1.49)	19.41 (4.26)	<0.0001
CDR-SOB	0.16 (0.37)	5.72 (2.90)	<0.0001
Age - Education level correlation	−0.090 (*p* = 0.43)	0.021 (*p* = 0.85)	-
Sex - Education level correlation	−0.188 (*p* = 0.09)	−0.032 (*p* = 0.78)	-
CDR-SOB - Education level correlation	−0.177 (*p* = 0.12)	0.138 (*p* = 0.22)	-

Continuous variables are presented as mean (SD) except correlation values.

K-MMSE = Korean version of mini-mental status exam.

Pearson’s correlation coefficients for sex were computed by coding male as 0 and female as 1, as in^67^.
