# Supplementary material for: A Network Flow-based Analysis of Cognitive Reserve in Normal Ageing and Alzheimer’s Disease
Source: Sci Rep. 2015 May 20;5:10057. doi: 10.1038/srep10057 (PMC4438712; doi:10.1038/srep10057)
Supplement: Supplementary Information [file srep10057-s1.doc]

**Research Article**

**A Network Flow-based Analysis of Cognitive Reserve in Normal Ageing and Alzheimer’s Disease**

Sang Wook Yoo^1, 2^, Cheol E. Han^1^, Joseph S. Shin (formerly Sung Yong Shin) ^2, 7^,

Sang Won Seo^3^, Duk L. Na^3^, Marcus Kaiser^4, 5^, Yong Jeong^6*^, Joon-Kyung Seong^1*^

^1^ Department of Biomedical Engineering, Korea University, Seoul, Republic of Korea, ^2^ Department of Computer Science, KAIST, Daejeon, Republic of Korea, ^3^ Department of Neurology, Sungkyunkwan University School of Medicine, Samsung Medical Center, Seoul, Korea, ^4^ Department of Brain & Cognitive Sciences, Seoul National University, Seoul 151–747, South Korea, ^5^ Interdisciplinary Computing and Complex BioSystems Research Group, School of Computing Science, Newcastle University, Newcastle upon Tyne, NE1 7RU, UK, ^6^ Department of Bio and Brain Engineering, KAIST, Daejeon, Republic of Korea, ^7^ Handong Global University, Pohang, Republic of Korea

Corresponding author #1: Joon-Kyung Seong, PhD

Corresponding author’s address: Department of Biomedical Engineering, Korea University, Seoul, Republic of Korea

Corresponding author’s phone and fax: +82-2-940-2887, no fax

Corresponding author’s e-mail address: [jkseong@korea.ac.kr](mailto:jkseong@korea.ac.kr)

Corresponding author #2: Yong Jeong, MD, PhD

Corresponding author’s address: Department of Bio and Brain Engineering, KAIST, Daejeon, Republic of Korea

Corresponding author’s phone and fax: +82-42-350-4324, +82-42-350-4380

Corresponding author’s e-mail address: yong@kaist.ac.kr

**Supplementary Table 1** Representative nodes and their anatomic regions and degrees in the subnetwork of the NC and AD groups. (NC subnetwork: Fiber number threshold = 3, correlation threshold = 0.32, *p*-value = 0.026±0.004, cluster

| **NC** | | | **AD** | | |
| --- | --- | --- | --- | --- | --- |
| **Node** | **Anatomic region** | **Degree** | **Node** | **Anatomic region** | **Degree** |
| SupraMarginal_L | Parietal lobe | 55 | Frontal_Mid_L | Frontal lobe | 47 |
| Precentral_L | Central region | 8 | Temporal_Pole_Mid_R | Limbic lobe | 12 |
| Temporal_Mid_L | Temporal lobe | 2 | Angular_L | Parietal lobe | 12 |
| Temporal_Pole_Sup_L | Limbic lobe | 2 | Frontal_Med_Orb_R | Frontal lobe | 10 |
| Putamen_R | Subcortical | 2 | Frontal_Inf_Oper_R | Frontal lobe | 9 |
| Precuneus_R | Parietal lobe | 2 | ParaHippocampal_R | Limbic lobe | 6 |
| Precuneus_L | Parietal lobe | 2 | Cingulum_Mid_R | Limbic lobe | 6 |
| Fusiform_R | Occipital lobe | 2 | Temporal_Inf_R | Temporal lobe | 5 |
| Occipital_Sup_R | Occipital lobe | 2 | Occipital_Mid_R | Occipital lobe | 5 |
| Calcarine_R | Occipital lobe | 2 | Rolandic_Oper_L | Central region | 5 |

size = 63 / AD subnetwork: Fiber number threshold = 3, correlation threshold = -0.3, *p*-value = 0.041±0.006, cluster size = 92)

Nodes are listed in descending order by their degrees in the subnetwork.

**Supplementary Table 2** Representative connections showing significant difference in the correlation coefficients (correlation difference threshold = 0.6)

| **Region 1** | **Region 2** | **Correlation for**  **NC group** | **Correlation for**  **AD group** | **Correlation difference** |
| --- | --- | --- | --- | --- |
| Frontal_Med_Orb_R | SupraMarginal_L | 0.489 | -0.226 | 0.715 |
| Occipital_Mid_R | SupraMarginal_L | 0.521 | -0.158 | 0.678 |
| Insula_R | SupraMarginal_L | 0.391 | -0.262 | 0.654 |
| Frontal_Sup_R | SupraMarginal_L | 0.491 | -0.147 | 0.638 |
| SupraMarginal_L | Temporal_Pole_Sup_R | 0.451 | -0.185 | 0.636 |
| ParaHippocampal_L | SupraMarginal_L | 0.417 | -0.212 | 0.629 |
| Frontal_Sup_Medial_L | SupraMarginal_L | 0.415 | -0.203 | 0.618 |
| Occipital_Mid_L | SupraMarginal_L | 0.489 | -0.116 | 0.605 |
| Lingual_R | SupraMarginal_L | 0.489 | -0.113 | 0.602 |
| Frontal_Med_Orb_R | SupraMarginal_L | 0.489 | -0.226 | 0.715 |
| Occipital_Mid_R | SupraMarginal_L | 0.521 | -0.158 | 0.678 |
| Insula_R | SupraMarginal_L | 0.391 | -0.262 | 0.654 |
| Frontal_Sup_R | SupraMarginal_L | 0.491 | -0.147 | 0.638 |

Connections are listed in descending order by their correlation coefficient difference

**Supplementary Table 3** Cortical regions and sub-cortical structures in the AAL template

| **AAL Regions** | **Abbreviation** | **AAL Regions** | **Abbreviation** |
| --- | --- | --- | --- |
| Precentral gyrus (left) | Precentral_L | Cuneus (right) | Cuneus_R |
| Precentral gyrus (right) | Precentral_R | Lingual gyrus (left) | Lingual_L |
| Superior frontal gyrus, dorsolateral (left) | Frontal_Sup_L | Lingual gyrus (right) | Lingual_R |
| Superior frontal gyrus, dorsolateral (right) | Frontal_Sup_R | Superior occipital gyrus (left) | Occipital_Sup_L |
| Superior frontal gyrus, orbital part (left) | Frontal_Sup_Orb_L | Superior occipital gyrus (right) | Occipital_Sup_R |
| Superior frontal gyrus, orbital part (right) | Frontal_Sup_Orb_R | Middle occipital gyrus (left) | Occipital_Mid_L |
| Middle frontal gyrus (left) | Frontal_Mid_L | Middle occipital gyrus (right) | Occipital_Mid_R |
| Middle frontal gyrus (right) | Frontal_Mid_R | Inferior occipital gyrus (left) | Occipital_Inf_L |
| Middle frontal gyrus, orbital part (left) | Frontal_Mid_Orb_L | Inferior occipital gyrus (right) | Occipital_Inf_R |
| Middle frontal gyrus, orbital part (right) | Frontal_Mid_Orb_R | Fusiform gyrus (left) | Fusiform_L |
| Inferior frontal gyrus, opercular part (left) | Frontal_Inf_Oper_L | Fusiform gyrus (right) | Fusiform_R |
| Inferior frontal gyrus, opercular part (right) | Frontal_Inf_Oper_R | Postcentral gyrus (left) | Postcentral_L |
| Inferior frontal gyrus, triangular part (left) | Frontal_Inf_Tri_L | Postcentral gyrus (right) | Postcentral_R |
| Inferior frontal gyrus, triangular part (right) | Frontal_Inf_Tri_R | Superior parietal gyrus (left) | Parietal_Sup_L |
| Inferior frontal gyrus, orbital part (left) | Frontal_Inf_Orb_L | Superior parietal gyrus (right) | Parietal_Sup_R |
| Inferior frontal gyrus, orbital part (right) | Frontal_Inf_Orb_R | Inferior parietal, but supramarginal and  angular gyri (left) | Parietal_Inf_L |
| Rolandic operculum (left) | Rolandic_Oper_L | Inferior parietal, but supramarginal and  angular gyri (right) | Parietal_Inf_R |
| Rolandic operculum (right) | Rolandic_Oper_R | Supramarginal gyrus (left) | SupraMarginal_L |
| Supplementary motor area (left) | Supp_Motor_Area_L | Supramarginal gyrus (right) | SupraMarginal_R |
| Supplementary motor area (right) | Supp_Motor_Area_R | Angular gyrus (left) | Angular_L |
| Olfactory cortex (left) | Olfactory_L | Angular gyrus (right) | Angular_R |
| Olfactory cortex (right) | Olfactory_R | Precuneus (left) | Precuneus_L |
| Superior frontal gyrus, medial (left) | Frontal_Sup_Medial_L | Precuneus (right) | Precuneus_R |
| Superior frontal gyrus, medial (right) | Frontal_Sup_Medial_R | Paracentral lobule (left) | Paracentral_Lobule_L |
| Superior frontal gyrus, medial orbital (left) | Frontal_Med_Orb_L | Paracentral lobule (right) | Paracentral_Lobule_R |
| Superior frontal gyrus, medial orbital (right) | Frontal_Med_Orb_R | Caudate nucleus (left) | Caudate_L |
| Gyrus rectus (left) | Rectus_L | Caudate nucleus (right) | Caudate_R |
| Gyrus rectus (right) | Rectus_R | Lenticular nucleus, putamen (left) | Putamen_L |
| Insula (left) | Insula_L | Lenticular nucleus, putamen (right) | Putamen_R |
| Insula (right) | Insula_R | Lenticular nucleus, pallidum (left) | Pallidum_L |
| Anterior cingulate and paracingulate gyri (left) | Cingulum_Ant_L | Lenticular nucleus, pallidum (right) | Pallidum_R |
| Anterior cingulate and paracingulate gyri (right) | Cingulum_Ant_R | Thalamus (left) | Thalamus_L |
| Median cingulate and paracingulate gyri (left) | Cingulum_Mid_L | Thalamus (right) | Thalamus_R |
| Median cingulate and paracingulate gyri (right) | Cingulum_Mid_R | Heschl gyrus (left) | Heschl_L |
| Posterior cingulate gyrus (left) | Cingulum_Post_L | Heschl gyrus (right) | Heschl_R |
| Posterior cingulate gyrus (right) | Cingulum_Post_R | Superior temporal gyrus (left) | Temporal_Sup_L |
| Hippocampus (left) | Hippocampus_L | Superior temporal gyrus (right) | Temporal_Sup_R |
| Hippocampus (right) | Hippocampus_R | Temporal pole: superior temporal gyrus (left) | Temporal_Pole_Sup_L |
| Parahippocampal gyrus (left) | ParaHippocampal_L | Temporal pole: superior temporal gyrus (right) | Temporal_Pole_Sup_R |
| Parahippocampal gyrus (right) | ParaHippocampal_R | Middle temporal gyrus (left) | Temporal_Mid_L |
| Amygdala (left) | Amygdala_L | Middle temporal gyrus (right) | Temporal_Mid_R |
| Amygdala (right) | Amygdala_R | Temporal pole: middle temporal gyrus (left) | Temporal_Pole_Mid_L |
| Calcarine fissure and surrounding cortex (left) | Calcarine_L | Temporal pole: middle temporal gyrus (right) | Temporal_Pole_Mid_R |
| Calcarine fissure and surrounding cortex (right) | Calcarine_R | Inferior temporal gyrus (left) | Temporal_Inf_L |
| Cuneus (left) | Cuneus_L | Inferior temporal gyrus (right) | Temporal_Inf_R |

**Supplementary Figure 1** An overview of the proposed approach.

a. An overview of the WM network construction procedure: First, the T1-weighted MRI for a subject is linearly registered (*L*) to the b0 image, and nonlinearly registered (*T*) to the ICBM152 T1 template in the MNI space. The AAL template regions in the MNI space are then transformed to the native T1 space with an inverse of the nonlinear transform (*T*^-1^), and then to the native diffusion space with the linear transform (*L*). Finally, the WM network is constructed with fiber tracts and AAL template regions in the native diffusion space.


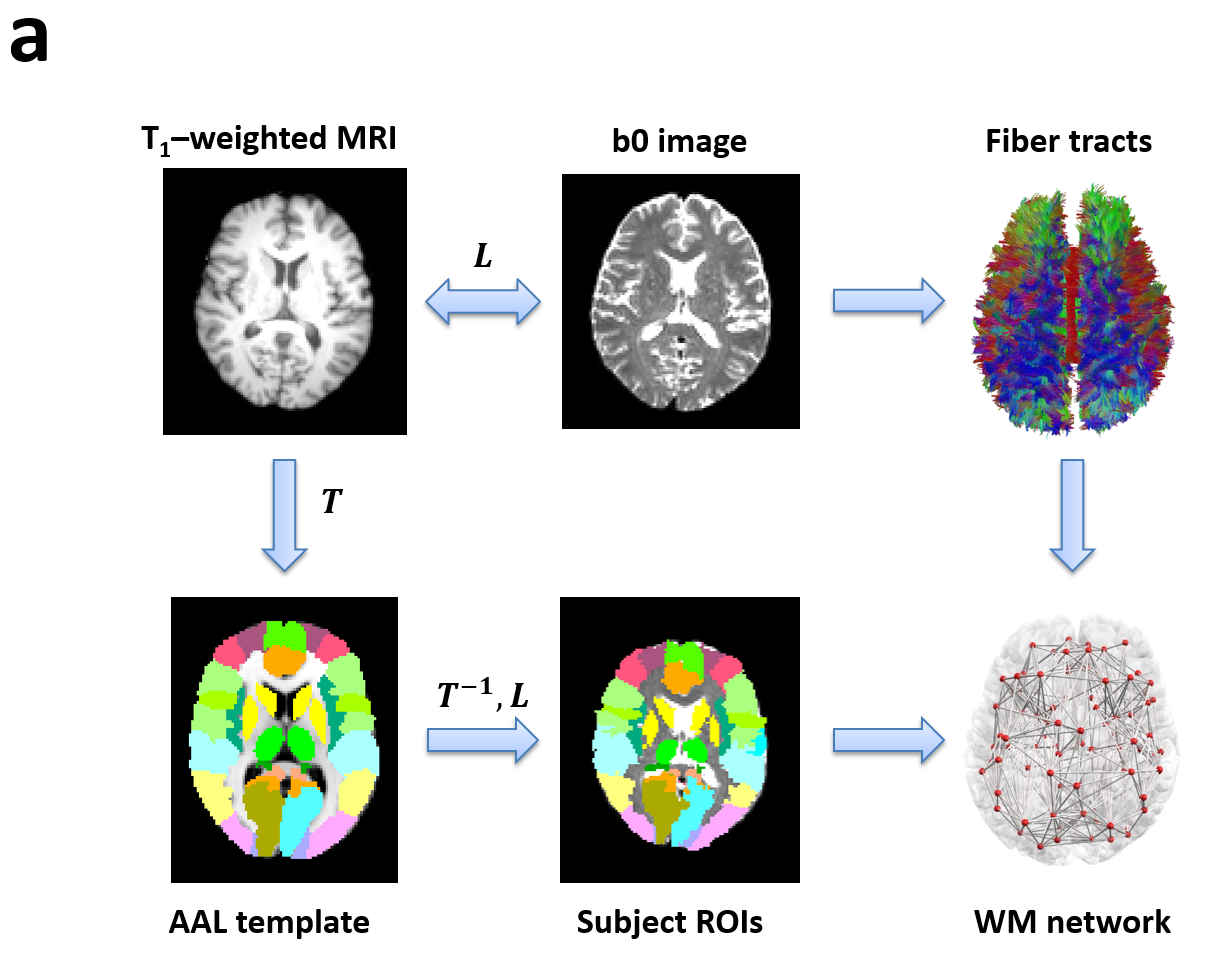


b. An overview of the WM network analysis.

**
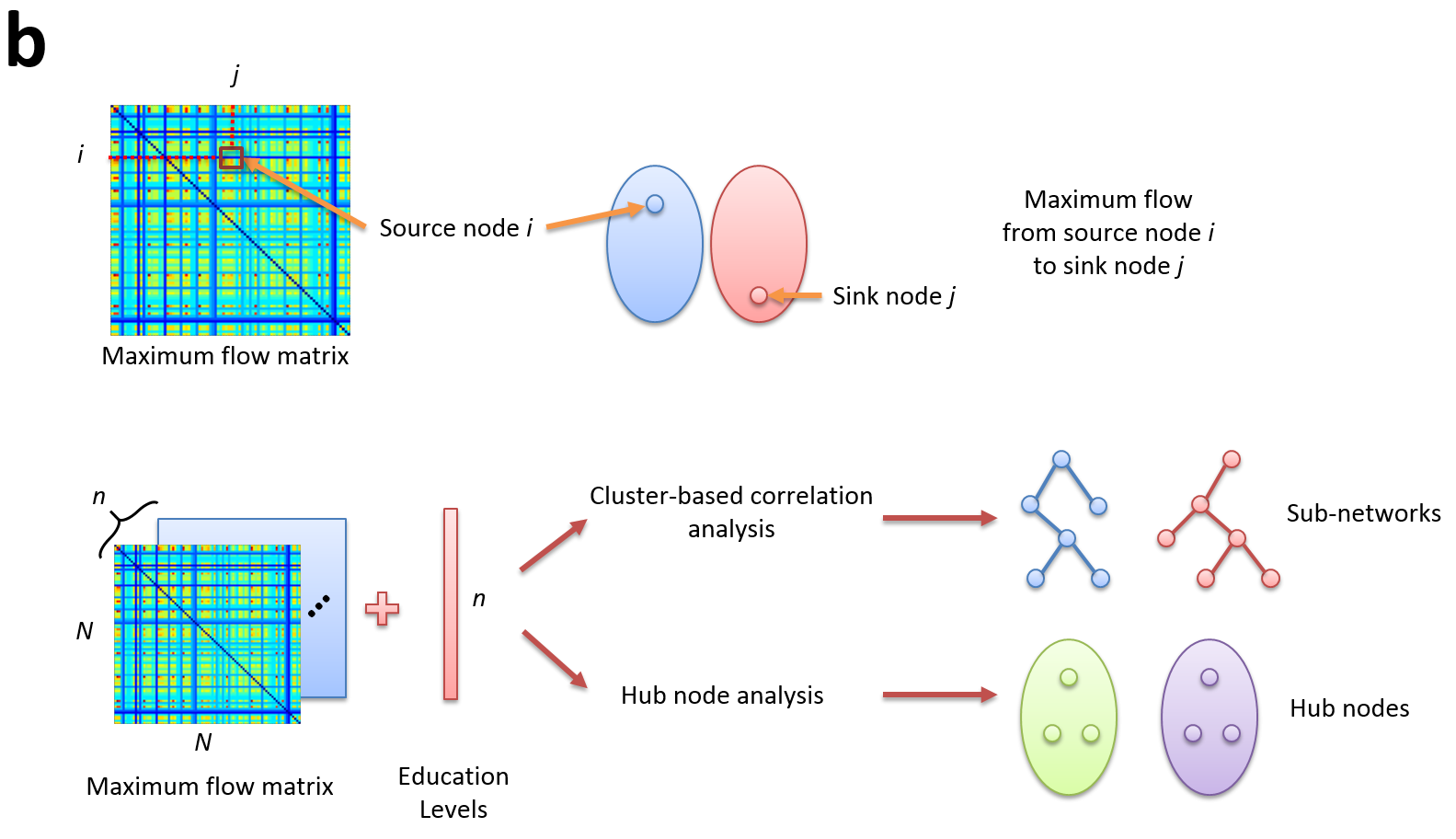
**

c. An illustrative example shows two binary graphs with the same number of edges but the different maximum flow values from node *i* to node *j*. There exist two edge-disjoint paths from node *i* to node *j* for the left graph, while four paths for the right one. Hence from the perspective of reliability or robustness in information flow, the right graph is more resistant to possible destruction of edges.


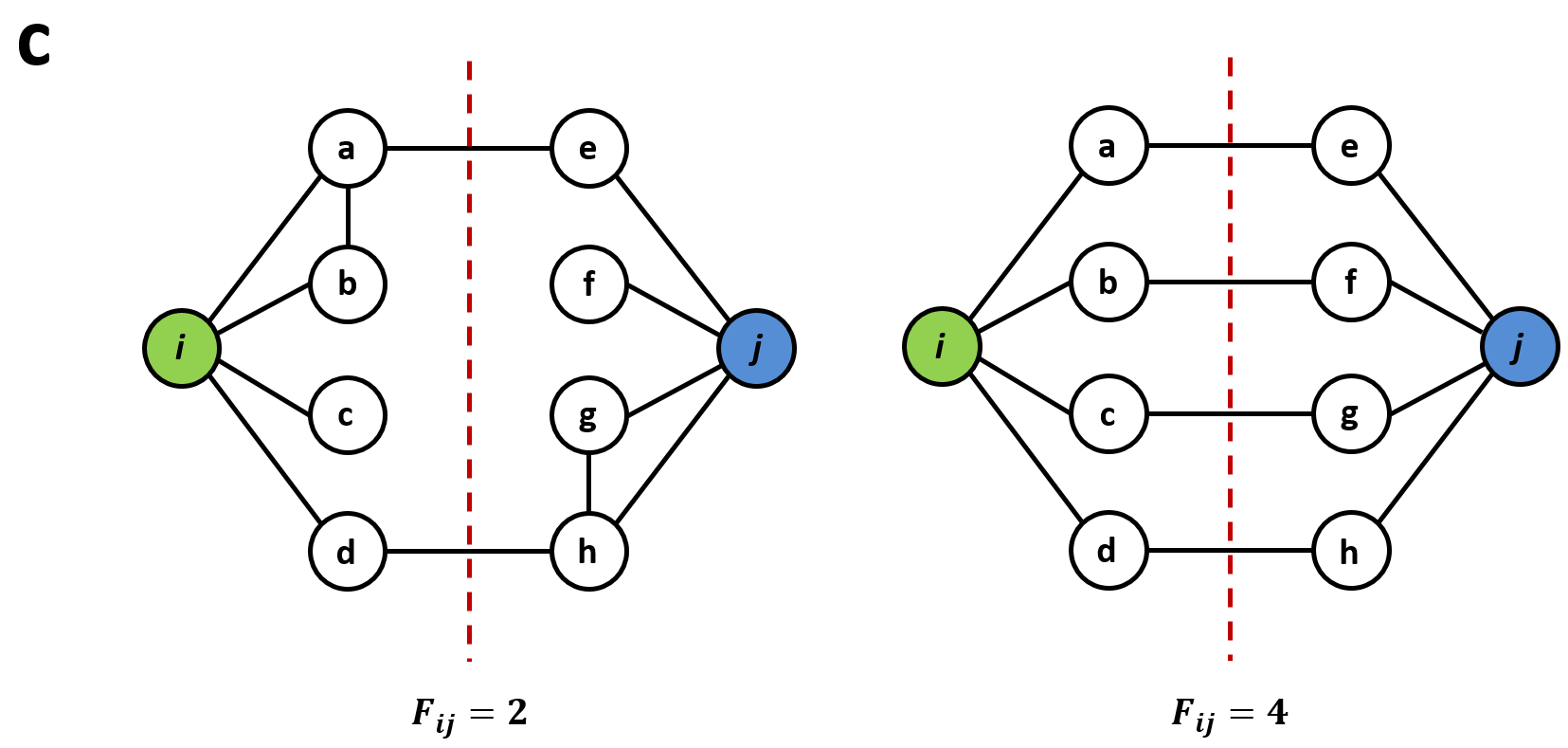


**Supplementary Methods**

**Maximum flow computation**

Given a WM network *G=* (*V, E, W*), the weight of each edge *a∊E* indicates its capacity *c*(*a*)*>0*, that is, the maximum rate at which a material can be transported along it. In our problem setting, *c*(*a*) is assigned with a binary value. In other words, the (*i*,*j*)th element of *W* is set to one if there is an edge from node *i* to node *j*; Otherwise, it is set to zero. Since the maximum flow is computed for a directed graph, we transform our WM network to the equivalent directed graph by admitting two opposite directed edges for each undirected one. The capacities of two opposite directed edges are set to that of the original undirected edge. The maximum flow from the source *s* to the sink *t* in the transformed graph is equal to that between the sink and the source in the original graph[^1^](#_ENREF_1).

In order to find the maximum flow value from the source *s* to the sink *t* in the WM network, we adopt the Ford-Fulkerson method[^1^](#_ENREF_1). The Ford-Fulkerson method finds the maximum flow in an iterative manner: Initially, the method starts with a zero flow value from the source *s* to the sink *t*. At each iteration, the method finds an “augmenting path” which is a path from the source *s* to the sink *t* along which more flow can be sent, with the flow not exceeding the capacity of any edge on the path. With an augmenting path found, the method computes the flow value of this path that saturates at least one of the edges along the path. Finally, the maximum flow value is computed by adding the flow values of the augmented paths until no more augmenting path can be found. The resulting maximum flow value is unique for any pair of the source *s* and the sink *t* in the network. Since the WM network has binary weights, the maximum flow value is equal to the number of edge-disjoint paths between source and sink nodes[^2^](#_ENREF_2). We use a publicly available implementation[^3^](#_ENREF_3) for maximum flow computation.

Along with the maximum flow value between node *s* and node *t*, we can also compute a set of bottleneck edges that restrict the flow value in the network. A cut in a network is a set of edges, denoted by *K,* that connect the node sets *S* and $\overline{S}$=*V - S*, where the source *s*∊*S* and the sink *t*∊$\overline{S}$. The capacity of a cut is the sum of the capacities of the edges that belongs to it. According to the maximum flow/minimum cut theorem[^1^](#_ENREF_1), the capacity of a minimum cut *K* in a network is equal to the maximum flow value. A maximum flow value saturates the edges in the minimum cut *K* which divide the nodes into the two sets *S* and$\overline{S}$. Thus, the set of edges in *K* can be regarded as the bottleneck of the material transmission in the network These edges can be obtained as a byproduct of the Ford-Fulkerson method[^1^](#_ENREF_1).

**Network hub analysis**

We further investigate the subnetworks identified in the Network analysis section to determine central brain regions that serve as hub nodes in a network. There have been many methods proposed for measuring centrality of a node based on several graph theoretic concepts, including the degree of a node, the degree of “betweenness”[^4^](#_ENREF_4) or “closeness”[^5^](#_ENREF_5) of a node within the overall network architecture. The closeness centrality of a node is calculated as the inverse of the average distance from the node to all other nodes in the network[^6^](#_ENREF_6). Similarly, the betweenness centrality of a node is defined as the fraction of shortest paths between any pair of nodes that travel through the node[^4^](#_ENREF_4). In our study, we employ all the three measures listed above for network core analysis. Our aim was to analyze the core of the identified subnetworks instead of the original WM networks. Since the subnetwork in our analysis is represented by a symmetric binary matrix, the nodal degree of the node *i* is the number of edges connected to the node. In order to calculate betweenness centrality and closeness centrality, the lengths of all shortest paths between any pair of nodes are computed by employing the Dijkstra’s algorithm[^7^](#_ENREF_7). We compute the mean and standard deviation of each measure. Then, we define the network hub as the node with the value greater than their sum. For each of the three measures, we determine a set of network hubs, separately.

REFERENCES

1. Ford LR, Fulkerson DR. *Flows in networks*. (Princeton University Press, 1962).

2. Wilson RJ. *Introduction to graph theory*. (Academic Press, 1972).

3. Boykov Y, Kolmogorov V. An experimental comparison of min-cut/max-flow algorithms for energy minimization in vision. *IEEE Trans Pattern Anal Mach Intell* **26**, 1124-1137 (2004).

4. Freeman LC. Set of Measures of Centrality Based on Betweenness. *Sociometry* **40**, 35-41 (1977).

5. Freeman LC. Centrality in Social Networks Conceptual Clarification. *Social Networks* **1**, 215-239 (1979).

6. Sporns O, Honey CJ, Kotter R. Identification and Classification of Hubs in Brain Networks. *Plos One* **2**, (2007).

7. Dijkstra EW. A Note on Two Problems in Connextion with Graphs. *Numerische Mathematik* **1**, 269-271 (1959).
